# Inflammaging markers characteristic of advanced age show similar levels with frailty and dependency

**DOI:** 10.1038/s41598-021-83991-7

**Published:** 2021-02-23

**Authors:** Ainhoa Alberro, Andrea Iribarren-Lopez, Matías Sáenz-Cuesta, Ander Matheu, Itziar Vergara, David Otaegui

**Affiliations:** 1 Biodonostia Health Research Institute, Multiple Sclerosis Group, San Sebastian, Spain; 2 Biodonostia Health Research Institute, Cellular Oncology Group, San Sebastian, Spain; 3 CIBER de Fragilidad y Envejecimiento Saludable (CIBERfes), Madrid, Spain; 4 IKERBASQUE, Basque Foundation for Science, Bilbao, Spain; 5 Biodonostia Health Research Institute, Primary Care Unit, San Sebastian, Spain; 6 Health Services Research on Chronic Patients Network (REDISSEC), Madrid, Spain; 7 Spanish Network of Multiple Sclerosis, Barcelona, Spain

**Keywords:** Cytokines, Biomarkers, Geriatrics

## Abstract

The improvement of life quality and medical advances has resulted in increased life expectancy. Despite this, health status commonly worsens in the last years of life. Frailty is an intermediate and reversible state that often precedes dependency and therefore, its identification may be essential to prevent dependency. However, there is no consensus on the best tools to identify frailty. In this sense, diverse molecules have been proposed as potential biomarkers. Some investigations pointed to an increased chronic inflammation or inflammaging with frailty, while others did not report such differences. In this work, we evaluated the circulating concentration of the inflammaging markers in adults and older adults (aged over 70 years) by ELISA and Luminex techniques. The Barthel Index was applied for the evaluation of dependency and Timed up-and-go, Gait Speed, Short Physical Performance Battery, Tilburg Frailty Indicator and Gerontopole Frailty Screening Tool were used for the identification of frailty. CRP, TNF-α, IL-6 and albumin concentrations were measured, and we found that elevated inflammation is present in older adults, while no differences with frailty and dependency were reported. Our results were consistent for all the evaluated frailty scales, highlighting the need to reconsider increased inflammation as a biomarker of frailty.

## Introduction

Life expectancy has increased notably in the last decades, but in most of the cases the last years of life are accompanied by comorbidities, disability and dependency^1^. These problems worsen the quality of life, increase the risk of hospitalization and institutionalization, and consequently, social and healthcare spending. Disability and dependency are usually preceded by frailty. Frailty is an intermediate and reversible state that often precedes dependency, characterised by a reduced functional reserve, impaired adaptive capacity across multiple physiological systems and increased vulnerability to stressors^2^. The accentuated vulnerability results in high risk of negative outcomes, such as falls, fractures, infections, disability, hospitalization, and death^3^. The opposite situation to frailty is termed robustness. An older adult is classified as robust when her/his functional capacity is conserved, and besides, phenotypic stability (or performance) is also maintained after the occurrence of a clinical stressor^4^. Importantly, frailty is the main risk factor for the development of disability among the community-dwelling older adults and can precede the deleterious outcomes by several years^5^. Therefore, the identification of frail individuals and the consequent interventions are key points for preventing dependency. However, frailty is a heterogeneous state comprising physical, psychological and cognitive impairment, and even if it has been widely studied for decades, no consensus has been reached on its definition and on the best tools to identify frailty.

Regarding the concept of frailty, a work by Rodríguez-Mañas and colleagues gathered the definitions of experts in the field and presented a list of accepted statements that define frailty^6^. This list included aspects of physical performance, nutritional status, mental health, and cognition. However, they concluded that, even if some concepts of frailty are widely agreed, there is no consensus on an operational definition of frailty. Despite the lack of a complete definition, as mentioned before, frailty results in an increased risk of developing dependence and it is generally accepted as a reversible state. Indeed, pharmacological, nutritional and physical interventions have been proposed to recover robustness^7^. Taken together, the main objective of frailty identification tools is to detect a person when she/he is at risk of developing dependence and intervene to prevent negative outcomes.

Several tests based on clinical and functional measures are applied in primary care services, but they evaluate different aspects of frailty and, therefore, the prevalence of frailty varies widely based on the test aplpied^8,9^. Aiming to complement these tests and to understand the biology of frailty, research on biomarkers of frailty is being conducted^10^. Distinct sources of biomarkers, including endocrine, inflammatory, metabolic, genetic and epigenetic markers among others have been proposed, with controversial results^11^. Hence, it is essential to continue investigating new potential biomarkers that could help the identification of frailty. In this sense, blood is an interesting biofluid for the search, as it is accessible, it can be processed and stored easily and it is routinely obtained at clinical settings. Similarly, the measurement of circulating inflammatory markers in blood is regularly performed and does not require complex or expensive procedures, so it is a promising source of biomarkers.

Indeed, studies have long shown that inflammatory markers are increased in aged individuals. The systemic and chronic low-grade inflammation observed in advanced age is generally referred to as inflammaging. Inflammaging is considered as a “sterile” inflammatory state, as it is present even in the absence of overt infection. Besides, it has been reported that inflammaging is a significant risk factor for morbidity and mortality in aged individuals^12^. There are several factors that can contribute to the increased concentration of inflammatory molecules with aging. The production of inflammatory mediators can be driven by damaged cells or debris that are not properly eliminated or by the increased number of senescent cells that accumulate with age and secrete a high amount of inflammatory molecules^13^.

Cytokines are one of the major regulators of inflammation. These small proteins are secreted by a wide range of cell types and they can promote or inhibit immune responses. A comprehensive work published by Minciullo and collaborators reviewed the role of proinflammatory and anti-inflammatory cytokines in aging and longevity^14^. Interestingly, the balance between the promoters and inhibitors of immune responses has been related to healthy aging and longevity. On the contrary, the destabilization of the system and the increase of proinflammatory cytokines results in inflammaging.

Among inflammaging, the most widely studied feature is the circulating concentration of interleukin-6 (IL-6). The concentration of this interleukin is normally low (or non-detectable) in healthy adults, while elevated levels of IL-6 have been reported in the older adults, with increasing concentrations in the very old^14,15^. Moreover, elevated IL-6 has also been associated with disability and mortality in older adults^16,17^. Other inflammatory mediators such as tumour necrosis factor alpha (TNF-α) and C-reactive protein (CRP) have also been investigated by many authors, and their concentration have also been found to be elevated in advanced age^18–20^.

The link between chronic inflammation and frailty has been previously investigated. The concentration of inflammatory mediators in circulation has been measured to test whether proinflammatory molecules are especially increased in frail individuals when compared to robusts. An elevated concentration of IL-6, TNF-α and CRP, among others, have been reported in most of the cohorts in frail aged participants^19,21–25^, while there are other studies that did not find significant differences between robust and frail individuals^26–28^. Another circulating molecule related to inflammation and proposed as a frailty biomarker is albumin. The rate of albumin synthesis is affected by both nutrition and inflammation, and inflammation alone is associated with a greater catabolic rate of albumin. Decreased albumin levels have been proposed as a risk factor for frailty, but similar to the above-mentioned inflammatory markers, there is no consensus on its validity^29,30^.

The aim of our study is to investigate the validity of inflammatory mediators as biomarkers that could complement the functional and clinical evaluation of older adults for the identification of frailty. To that end, we first compared the concentration of the above cited molecules between adults and older adults, and then, based on the frailty classification of older adults, evaluated whether these molecules show different levels with frailty and dependency in our cohorts.

## Materials and methods

### Participants and frailty classification

Samples from older adult donors (n = 199) and from healthy adults (n = 57) were used for the present study. We obtained the samples in collaboration with the Primary Care Unit of Biodonostia Health Research Institute and the Basque Biobank (www.biobancovasco.org). All participants are from the province of Gipuzkoa (Basque Country, Spain). Participants completed a questionnaire and donors with acute illness were excluded. The study was approved by the Donostia University Hospital’s ethics committee and all donors provided written informed consent before blood sampling. All the methods performed in this study were carried out in accordance with the relevant guidelines.

For the adult group, donors aged 20–49 years and with no chronic diseases or syndromes were enrolled. Frailty status of older adults was assessed by primary care services. Two different inclusion criteria and classification strategies were applied. The first cohort (Cohort 1) was composed of individuals aged 70 or over, community-dwelling and autonomous (Barthel > 90)^31^. The participants that fulfilled these criteria were enrolled, and the incidence of frailty was evaluated by several test. Frailty was assessed by Timed up-and-go (TUG)^32^, Gait Speed (GS)^33^, Short Physical Performance Battery (SPPB)^34^, Tilburg Frailty Indicator (TFI)^35^ and Gerontopole Frailty Screening Tool (GFST)^36^. The second cohort (Cohort 2) was completed with community-dwelling participants aged 70 or over. All participants of Cohort 2 were assessed by Barthel and TUG tests. Individuals with a Barthel score ≤ 90 were classified as non-autonomous, and among the older adults that obtained a Barthel > 90, robust and frails were identified by TUG.. A brief description of the applied tests is presented in Table 1 and a detailed description of the cohorts is presented in Table 2.

**Table 1:** Brief description of the dependency and frailty tests applied for the classification of older adults.

Assessment test	Description
Barthel index (Barthel)^31^	A multiparametric test measuring the performance in activities of daily living and mobility
Timed up-and-go (TUG)^32^	The time needed to stand up from a chair, walk 3 m, turn around, walk back and sit down, with the help of their usual walking aid, if any
Gait speed (GS)^33^	Expressed in meters per second (m/s). Participants were asked to walk at their usual pace. The test was performed twice, and GS was calculated based on the shorter time
Short physical performance battery (SPPB)^34^	A functional capacity test composed of gait speed, test of balance and time needed to stand up from a chair 5 consecutive times
Tilburg frailty indicator (TFI)^35^	A user-friendly questionnaire based on a multidimensional approach. It is composed of a physical, a psychological and a social domain
Gerontopole frailty screening tool (GFST)^36^	Based on clinical judgement. 6 yes/no questions that help the physician to evaluate the existence of frailty

The assessment was performed by primary care professionals.

**Table 2:** Characteristics of the studied population.

	Older adults		Adults
	Cohort 1	Cohort 2	
**Participants (n)**	111	88	57
**Sex**			
Female (%)	65 (58.56%)	56 (63.64%)	30 (52.63%)
Male (%)	46 (41.44%)	32 (36.36%)	27 (47.37%)
**Age (in years)**			
Inclusion criteria	> 70	> 70	20–49
Mean (SD)	79.77 (4.00)	76.98 (5.84)	33.51 (7.17)
**Dependency assessment (Barthel)**			
Inclusion criteria	> 90	–	
Autonomous (%)	111 (100%)	75 (85.23%)	
Non-autonomous (%)	0 (0%)	13 (14.77%)	
**Frailty assessment**			
** Timed up-and-go (TUG)**			
Robust (%)	70 (63.06%)	42 (47.73%)	
Frail (%)	41 (36.94%)	46 (52.27%)	
** Gait speed (GS)**			
Robust (%)	82 (73.87%)		
Frail (%)	29 (26.13%)		
** Short physical performance battery (SPPB)**			
Robust (%)	49 (44.14%)		
Frail (%)	62 (55.86%)		
** Tilburg frailty indicator (TFI)**			
Robust (%)	55 (49.55%)		
Frail (%)	56 (50.45%)		
** Gerontopole frailty screening tool (GFST)**			
Robust (%)	78 (70.27%)		
Frail (%)	27 (24.32%)		
No data (%)	6 (5.41%)		

### Blood sampling

Peripheral blood was collected by experienced nurses by venipuncture with a 21-gage needle in 8 ml serum separator tubes and 4 ml EDTA tubes (Vacutainer, BD Biosciences) and directly deposited in the Basque Biobank for their processing and storage. Serum separator tubes were allowed to clot for 30 min and centrifuged at 1258*g* for 20 min to recover serum from the supernatant. EDTA tubes were kept upright and centrifuged at 1258*g* for 20 min to recover plasma. The obtained serum and plasma samples were aliquoted and stored at − 80 °C. Corresponding request forms were fulfilled to obtain the samples from the Basque Biobank (www.biobancovasco.org).

### ELISA

CRP concentration in plasma was measured with Quantikine ELISA (Catalog# DCRP00, R&D, Biotechne) following the manufacturer’s instructions. Plasma samples of the first cohort were diluted 1:150 to fit the standard curve of the kit.

To test serum samples CRP, TNF-α and IL-6 concentration were measured with Quantikine ELISAs (Catalog# DCRP00, HSTA00E and HS600B respectively, R&D, Biotechne) and albumin concentration with an ELISA kit (Catalog# EHALB, Invitrogen, Thermo Fisher Scientific) following the manufacturer’s instructions. Serum samples of the second cohort were diluted to fit the standard curves of each kit: diluted 1:100 for CRP, undiluted for TNF-α, undiluted for IL-6 and diluted 1:500,000 for albumin. For all the ELISA experiments samples were tested in duplicate and results with a CV > 20% were discarded.

### Luminex

A panel of 6 interleukins was designed for Luminex measurement: IL-6, IL-10, IL-2, IL-1β, IL-1Ra and TNF-α. The Milliplex Map #HCYTOMAG-60 K kit (Merck Millipore) was used. Manufacturer’s instructions were followed and plasma samples from the first cohort were assayed undiluted. The obtained results were not conclusive, as only the measurements of TNF-α were above the first point of the standard curve. We performed a second assay with the same kit and obtained similar results. Following the recommendations of the manufacturer to solve this issue, we repeated the assays using the high sensitivity kit #HSTCMAG-28SK (Merck Millipore) provided by the manufacturer, but most of the samples were still non-detectable. Lastly, we also tried a high sensitivity Luminex kit from another brand, #FCSTM09-04 (R&D, Biotechne) for IL-6, IL-10, IL-2 and IL-1β analytes. With this kit the measurement of the analytes was also non-detectable in many samples (65/160). In consequence, we decided not to measure more plasma samples with the Luminex technique and we analysed only the results from TNF-α, the only analyte that obtained detectable and reliable results. Samples were tested in duplicate and all results had a CV < 20%.

### Statistical analysis

Statistically significant differences between the study groups and correlations between variables were tested with GraphPad Prism version 6.01 for Windows (GraphPad Software, www.graphpad.com). D’Agostino-Pearson normality test was applied and non-Gaussian distribution was confirmed for all samples. Consequently, Mann–Whitney tests were applied to evaluate differences between two study groups. For correlation analysis, Spearman coefficient was calculated. The statistically significant differences are presented as: *p < 0.05, **p < 0.01, ***p < 0.001 and ****p < 0.0001.

## Results

### Inflammation in plasma increases with age but not with frailty

**Figure 1:**
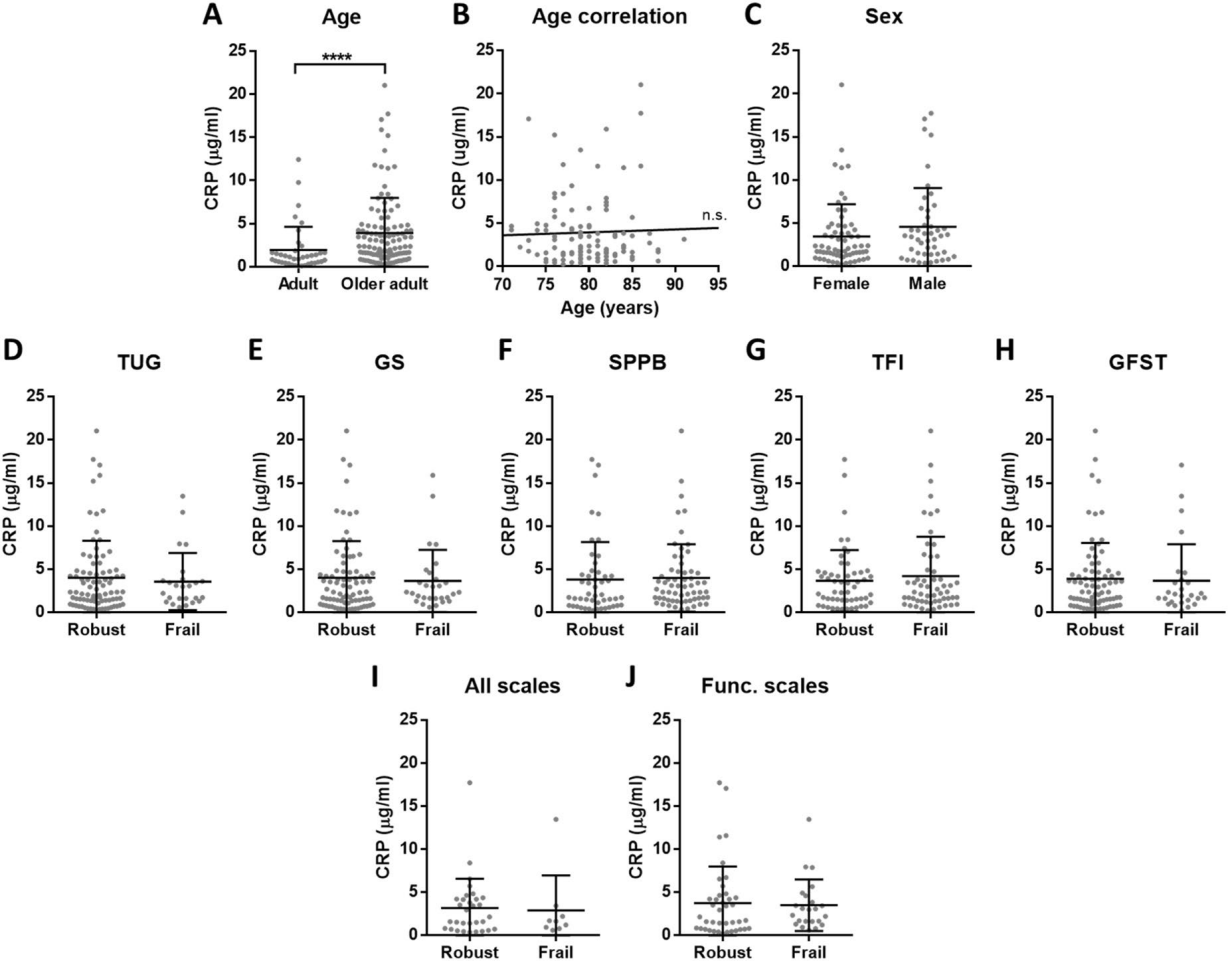
Concentration of CRP in plasma. (**A**) There is elevated CRP (p < 0.0001****) in older adults (n = 111) compared to adults (n = 38). (**B**) Among older adults, CRP concentration has no correlation with age and (**C**) there is no significant difference between females and males. (**D–H**) No differences in CRP levels between robust and frail individuals were found for the 5 analysed frailty scales. (**I**) We also compared the individuals classified as robust or frail with all the tests (n = 40) or (**J**) with the 3 functional scales (TUG, GS and SPPB) (n = 63), but no differences were reported.

**Figure 2:**
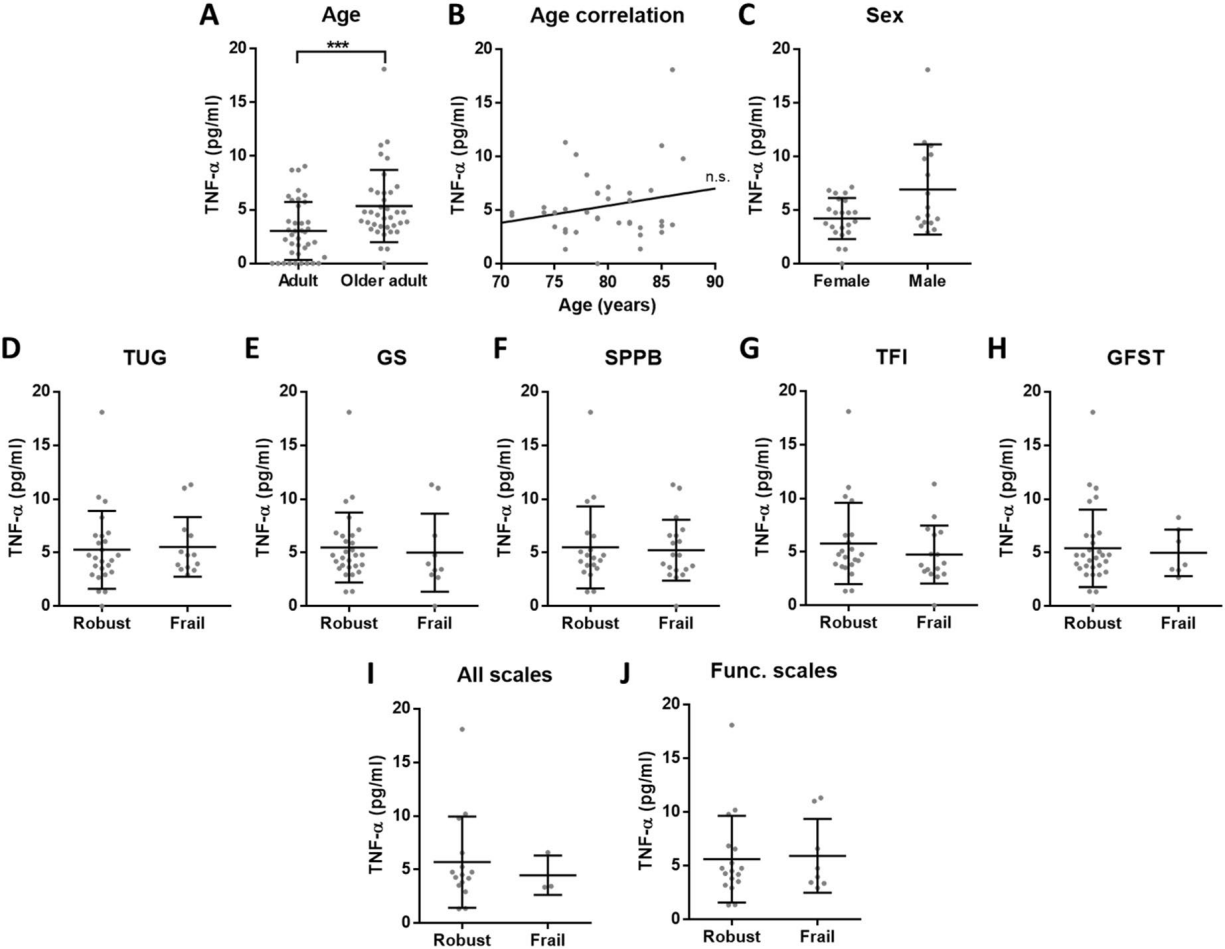
Concentration of TNF-α in plasma. (**A**) There is elevated TNF-α (p = 0.0006***) in older adults (n = 37) compared to adults (n = 39). (**B**) Among older adults, TNF-α concentration has no correlation with age and (**C**) there is no significant difference between females and males. (**D–H**) No differences in TNF-α levels between robust and frail individuals were found for the 5 analysed frailty scales. (**I**) We also compared the individuals classified as robust or frail with all the tests (n = 18) or (**J**) with the 3 functional scales (TUG, GS and SPPB) (n = 25), but no differences were reported.

To evaluate the concentration of inflammatory molecules, CRP and TNF-α were measured in plasma samples of adults and older adults from Cohort 1. First, results from older adults were compared to healthy adults, and we confirmed an increased concentration of both CRP and TNF-α in aged individuals (Figs. 1A, 2A). Then, the correlation between age and inflammatory markers was evaluated for older adults, but no significant correlations were found (Figs. 1B, 2B). Similarly, no differences were found based on sex (Figs. 1C, 2C). Finally, the concentration of the two inflammatory markers was compared between robust and frail participants. As frailty was evaluated by 5 different tests—TUG, GS, SPPB, TFI and GFST—the classification of each frailty scale was considered, and no significant differences were reported (Figs. 1D–H, 2D–H). We also investigated whether the participants that are classified as robust or frail for all the tested scales (n = 40) show elevated inflammation, but no differences were found (Figs. 1I, 2I). In the last approach, the older adults that are classified as robust or frail for the 3 scales that evaluate the functional status (TUG, GS and SPPB, n = 63) were brought into comparison, and as for the previous analyses, no differences were found (Figs. 1J, 2J). Given the continuous nature of the TUG and GS, we also checked for correlations between these tests and the inflammatory markers, but there were no significant correlations (Supplementary Table S1).

### Inflammaging levels are equal in serum of robust, frail and non-autonomous older adults

Next, we conducted a second experiment to confirm and complement our results. To exclude possible differences between plasma and serum, serum samples were measured in robust and frail older adults. Besides, we also included non-autonomous donors and investigated whether the concentration of inflammatory molecules could be an indicator of dependency. For this purpose, the samples from Cohort 2 of older adults were evaluated. For the characterization of inflammatory markers in serum, we studied the previously measured CRP and TNF-α, as well as IL-6 and albumin.

**Figure 3:**
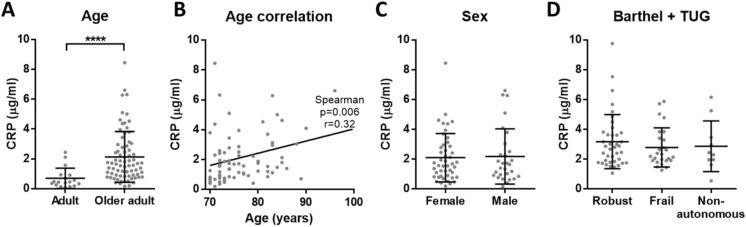
Concentration of CRP in serum. (**A**) There is elevated CRP (p < 0.0001****) in older adults (n = 75) when compared to adults (n = 18). (**B**) Among older adults, CRP concentration has a positive correlation with age (p = 0.006**, r = 0.32 and 95% confidence interval 0.088–0.511) and (**C**) there is no significant difference between females and males. (**D**) Based on Barthel and TUG scales, no differences in CRP levels between robust, frail and non-autonomous individuals were found.

**Figure 4:**
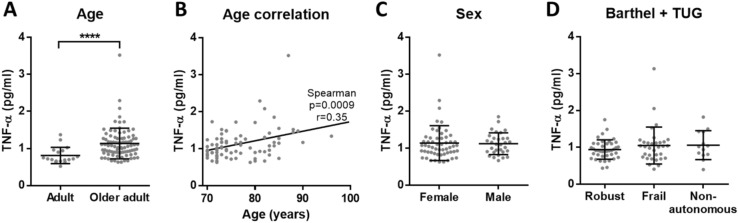
Concentration of TNF-α in serum. (**A**) There is elevated TNF-α concentration (p < 0.0001****) in older adults (n = 87) compared to adults (n = 18). (**B**) Among older adults, serum TNF-α concentration has a positive correlation with age (p = 0.0009***, r = 0.35 and 95% confidence interval 0.1425 to 0.5255) and (**C**) there is no significant difference between females and males. (**D**) Based on Barthel and TUG scales, there are no differences in TNF-α concentration between robust, frail and non-autonomous individuals.

**Figure 5:**
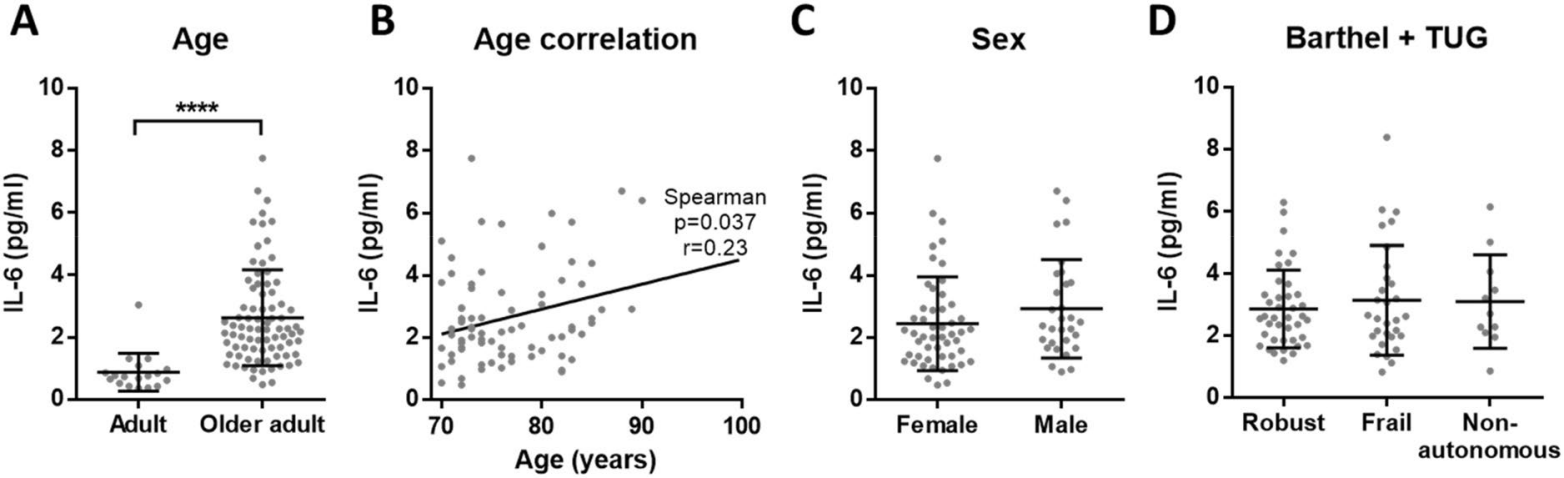
Concentration of IL-6 in serum. (**A**) There is elevated IL-6 (p < 0.0001****) in older adults (n = 81) when compared to adults (n = 18). (**B**) Among older adults, serum IL-6 concentration has a positive correlation with age (p = 0.037*, r = 0.23 and 95% confidence interval 0.01–0.43) and (**C**) there is no significant difference between females and males. (**D**) Based on Barthel and TUG scales, no differences in IL-6 levels between robust, frail and non-autonomous individuals were reported.

**Figure 6:**
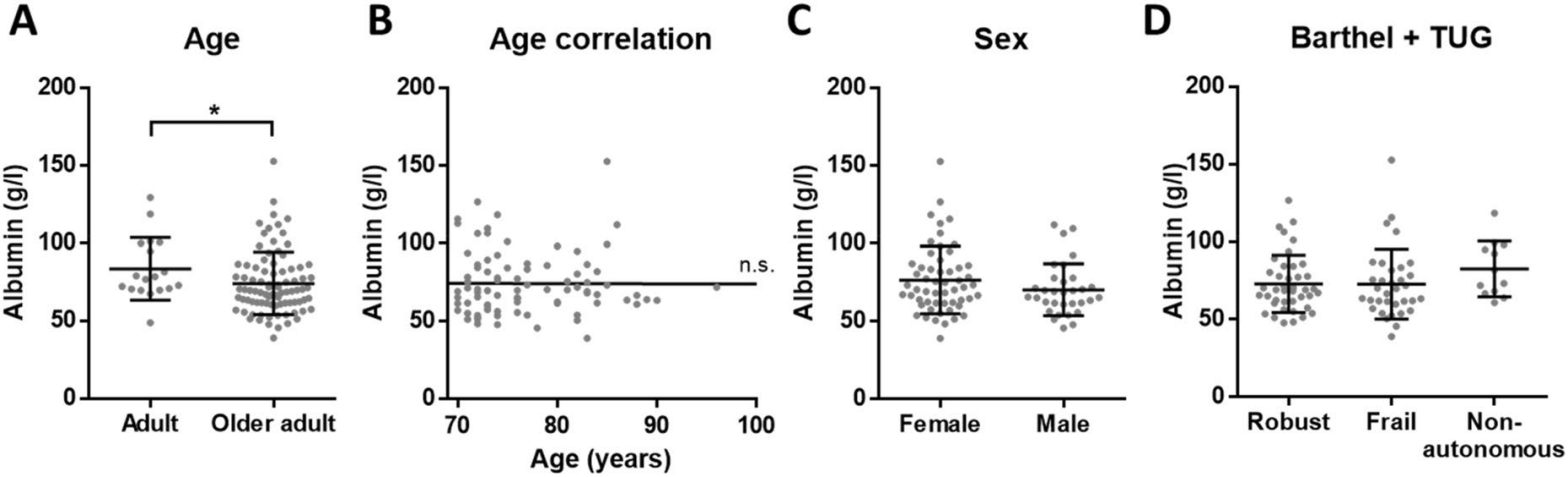
Concentration of albumin in serum. (**A**) There are reduced levels of albumin (p = 0.0242*) in older adults (n = 87) when compared to adults (n = 18). (**B**) Among aged participants, albumin concentration has no correlation with age and (**C**) there is no significant difference between females and males. (**D**) Based on Barthel and TUG scales, no differences in albumin concentration between robust, frail and non-autonomous individuals were found.

We obtained the same results as in plasma, confirming that there is an elevated chronic inflammation in older adults when compared to adults: increased CRP, TNF-α and IL-6, while reduced albumin (Figs. 3A, 4A, 5A and 6A). Moreover, CRP, TNF-α and IL-6 showed a significant positive correlation with age among older adults (Figs. 3B, 4B and 5B). On the other hand, no correlation with age was found for albumin (Fig. 6B). Regarding the sex, no differences were found for any of the analytes (Figs. 3C, 4C, 5C and 6C). Finally, based on the data of Barthel and TUG scales, the comparison between the different dependency statuses of participants was conducted. We found no significant differences for CRP, TNF-α, IL-6 and albumin concentration in serum between robust, frail and non-autonomous older adults (Figs. 3D, 4D, 5D and 6D). As in the previous experiment, given the continuous nature of the TUG test, we checked for correlations between TUG and the inflammatory markers. No correlations were found for CRP and albumin, while there was a significant positive correlation for TNF-α and IL-6 with TUG (Supplementary Table S1 and Fig. S1).

## Discussion

Inflammaging is one of the main biological characteristics of human aging. This term was proposed in 2000 by Franceschi et al.^12^, although a work showing the accumulation of inflammation with age and its relation to mortality was already published in 1991 by Mooradian et al.^37^. Since this term was introduced, many works have investigated the relationship between inflammatory markers and aging, dependency and mortality. However, the obtained results are diverse and many times discordant, so no consensus has been reached^14,19^.

The studies investigating the potential role of molecules linked to inflammation as biomarkers of frailty encounter the same problem. Many works have been carried out, but no clear association has been found, and as it is discussed below, some found increased concentrations of proinflammatory markers in frail subjects, whereas others did not report any significant differences. It should be considered that the study designs, the applied techniques and the characteristics of included participants are distinct in each investigation. Moreover, the published studies have been carried out in different countries, hence distinctive genetic and environmental aspects should be considered. In addition, the tests employed for frailty assessment evaluate the status of participants based on different aspects, and therefore, the same person can be considered frail based on one scale and robust based on another one. Indeed, there are frailty tests that focus on clinical aspects such as the GFST^36^, while others also consider the psychological and social domains like the TFI^35^ and the Edmonton Frail Scale^38^ and other ones to the functional performance, which include the TUG, GS and SPPB^32–34^, among others. Moreover, there are many other approaches to identify frailty. For instance, the one proposed by Linda Fried and collaborators in 2001^39^, as well as the Frailty Index^40,41^, have shown good predictive values and have been widely applied in research, while many geriatricians and researchers consider that they are complex tools that cannot be routinely applied in the everyday clinic. The diversity of tests and definitions shows the heterogeneity of the “frailty” term, which makes it even more difficult to identify a biological marker of the syndrome^6^.

We reliably measured CRP, TNF-α, IL-6 and albumin in our cohorts, that include aged subjects who have been evaluated with several frailty scales. In these cohorts, our results confirmed the presence of inflammaging by the increased low-grade inflammation in older adults when compared to adults. However, regarding frailty and dependency, none of the analysed molecules showed significant differences. Interestingly, we observed that adults had more similar circulating levels of the analysed inflammatory markers, while the concentrations measured for aged individuals were more heterogeneous (see A panels of the figures). This overall increased variability among older adults could be explained due to the complex and heterogeneous nature of aging^42^. Indeed, as pointed out by Franceschi and co-authors more than 20 years ago, the study on aging indicates that an increasing heterogeneity is a general phenomenon that unavoidably accompanies the aging process^43^.

Moreover, to try to overcome the above-mentioned issue of the heterogeneity between frailty tests, and taking advantage of the available data in Cohort 1, we also compared the concentrations of the inflammatory molecules in plasma between the participants that were classified as robust or frail for all the recorded frailty scales. Still, no differences were found when the participants that obtained the same classification for the 5 applied scales were considered. Additionally, and following recent recommendations of the WHO^44,45^, we focused on the results of the tests that evaluate functional performance (measured by GS, TUG and SPPB in this study), comparing the individuals that obtained the same results for those 3 frailty scales. Again, no differences were reported under these conditions. Besides, similar levels of inflammatory markers in serum were also reported in non-autonomous older adults. Therefore, we conclude that in our cohorts, robust, frail and non-autonomous individuals have no significant differences for the measured inflammatory markers. Notably, when associations between continuous frailty tests and inflammatory mediators were evaluated, a positive association between serum IL-6 and TUG score was found, and also between serum TNF-α and TUG, while the association with TNF-α was not reported in the case of plasma samples. This variation could be due to the limited number of plasma samples analysed as a result of technical problems, and/or due to concentration differences in serum and plasma or the different inclusion criteria of the two cohorts. Indeed, non-autonomous older adults were not included in Cohort 1, which probably reduced high scoring individuals at the TUG test. Hence, we consider that these associations should be further investigated in order to evaluate their validity.

To our knowledge, this is the first work studying inflammaging and frailty in the region of the Basque Country. Different results were previously reported in other regions of Spain: increased levels of CRP, TNF-α and IL-6 in frails were found in Galicia^46,47^, while no differences for IL-6, IL-8 and IL-10 and increased levels only for TNF-α were found in Granada^48^. These contrasting results were obtained even if the mentioned studies were carried out in the same country, and the three of them analysed plasma samples and used the Fried’s criteria to evaluate frailty. Interestingly, an exhaustive work published by Collerton and collaborators in 2012 (part of the Newcastle + 85 Study, England) in which both Fried’s criteria (n = 522) Frailty Index (n = 811) were used, reported positive associations of CRP, IL-6, and TNF-α with both frailty measures and an inverse association of albumin. They included community-dwelling and institutionalized older adults, but they did not state whether the analyses were carried in plasma or serum samples^23^. Remarkably, different results in both plasma and serum samples have also been reported. For instance, a work carried out in Texas found elevated IL-6 levels in plasma of frail donors evaluated by Fried’s criteria^24^, similar to the above-mentioned studies from Galicia, but in contrast to the work performed in Granada. Besides, a work from China recently reported significantly elevated serum concentrations of IL-6 (among other markers) in frail older adults (Fried’s criteria), while no differences were reported for CRP. Moreover, serum IL-6 levels were negatively correlated with both grip strength and gait speed^49^. In a different approach, a 10-year longitudinal study in community-dwelling older people in England, measured immune and endocrine markers in serum at baseline and evaluated their association with Fried frailty at follow-up, and found no association between specific pro-inflammatory cytokines (including IL-6) or CRP and frailty. Interestingly, other molecules were more promising, as higher baseline levels of differential white cell counts, lower levels of DHEAS and higher cortisol:DHEAS ratio were all significantly associated with increased odds of frailty at 10-year follow-up^50^.

Thus, the diversity of study approaches and outcomes becomes evident. Even if several works point to the use of inflammatory mediators as biomarkers of frailty^51^, there are also many other works that discourage this strategy. In the present study, we decided to study community-dwelling older adults and to apply several frailty assessment tools that are commonly used by clinicians, and found that inflammaging markers show similar levels with frailty and dependency.

In short, we think that the utility of these inflammatory molecules as cross-sectional biomarkers of frailty needs to be reconsidered, since a robust biomarker should be replicable. We believe that other approaches such as the development of longitudinal studies would be of great interest, as they enable the follow-up of participants as they age and frailty starts to arise, and the correlation with the levels of inflammatory markers could be evaluated. With this experimental setup, the changes in the concentration of inflammatory molecules could be measured in each participant, and it could be tested individually whether the progression of a specific molecule is related to frailty. Moreover, these circulating molecules are increased in a vast range of inflammatory or infectious conditions, so they could not be used as a single measure, and they should be applied in combination with other biomarkers that provide information about additional variables related to frailty, such as muscle loss or bone degeneration^11^.

## Supplementary Information

Supplementary Information
