# Supplementary material for: Inflammaging markers characteristic of advanced age show similar levels with frailty and dependency
Source: Sci Rep. 2021 Feb 23;11:4358. doi: 10.1038/s41598-021-83991-7 (PMC7902838; doi:10.1038/s41598-021-83991-7)
Supplement: Supplementary file 1 — Supplementary Information [file 41598_2021_83991_MOESM1_ESM.docx]

**Supplementary Information for**

**Inflammaging markers characteristic of advanced age show similar levels with frailty and dependency**

Ainhoa Alberro^1^, Andrea Iribarren-Lopez^1^, Matías Sáenz-Cuesta^1^, Ander Matheu^2,3,4^, Itziar Vergara^5,6^ and David Otaegui^1,7^*

^1^ Biodonostia Health Research Institute, Multiple Sclerosis Group, San Sebastian, Spain.

^2^ Biodonostia Health Research Institute, Cellular Oncology Group, San Sebastian, Spain.

^3^ CIBER de Fragilidad y Envejecimiento Saludable (CIBERfes), Madrid, Spain.

^4^ IKERBASQUE, Basque Foundation for Science, Bilbao, Spain.

^5^ Biodonostia Health Research Institute, Primary Care Unit, San Sebastian, Spain.

^6^ Health Services Research on Chronic Patients Network (REDISSEC), Madrid, Spain.

^7^ Spanish Network of Multiple Sclerosis, Barcelona, Spain.

**Table S1**. Spearman correlations between frailty assessment tests and inflammatory markers.

|  |  | **Timed up-and-go (TUG)** | |  | **Gait Speed (GS)** | |  |
| --- | --- | --- | --- | --- | --- | --- | --- |
|  | n | r | p |  | r | p |  |
| **Cohort 1** | CRP | 111 | 0.12 | 0.23 |  | -0.01 | 0.91 |
|  | TNF-α | 37 | 0.10 | 0.54 |  | -0.03 | 0.85 |
| **Cohort 2** | CRP | 74 | 0.11 | 0.36 |  |  |  |
|  | TNF-α | 84 | **0.42** | **<0.0001** |  |  |  |
|  | IL-6 | 79 | **0.26** | **0.0227** |  |  |  |
|  | Albumin | 85 | 0.04 | 0.73 |  |  |  |

Significant correlations are indicated in bold.


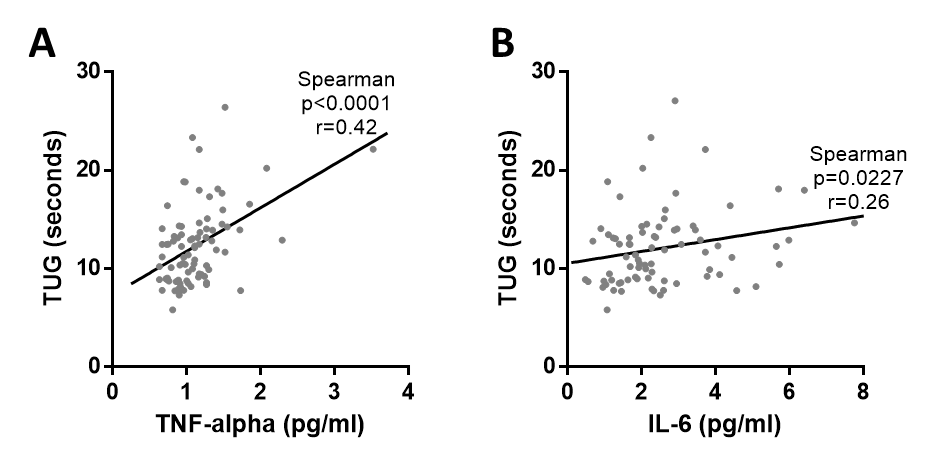


**Figure S1**. Significant correlations between frailty assessment tests and inflammatory markers. (**A**) Positive correlations between TNF-α and TUG (p<0.0001****, r=0.42 and 95% confidence interval 0.215 to 0.583) and (**B**) between IL-6 and TUG (p=0.0227*, r=0.26 and 95% confidence interval 0.030 to 0.457) were reported in Cohort 2.
